# *Salmonella enterica* from a soldier from the 1652 siege of Barcelona (Spain) supports historical transatlantic epidemic contacts

**DOI:** 10.1016/j.isci.2021.103021

**Published:** 2021-08-24

**Authors:** Toni de-Dios, Pablo Carrión, Iñigo Olalde, Laia Llovera Nadal, Esther Lizano, Dídac Pàmies, Tomas Marques-Bonet, François Balloux, Lucy van Dorp, Carles Lalueza-Fox

**Affiliations:** 1 Institute of Evolutionary Biology (CSIC-UPF), 08003 Barcelona, Spain; 2 Antequem. Arqueologia-Patrimoni Cultural, 08301 Mataró, Spain; 3 Catalan Institution of Research and Advanced Studies (ICREA), 08010 Barcelona, Spain; 4 CNAG-CRG, Centre for Genomic Regulation, Barcelona Institute of Science and Technology (BIST), 08028 Barcelona, Spain; 5 Institut Català de Paleontologia Miquel Crusafont, Universitat Autònoma de Barcelona, 08193 Cerdanyola del Vallès, Spain; 6 UCL Genetics Institute, University College London, London WC1E 6BT, UK

**Keywords:** Biological sciences, Genomic analysis, Genomics, Paleogenetics

## Abstract

Ancient pathogen genomics is an emerging field allowing reconstruction of past epidemics. The demise of post-contact American populations may, at least in part, have been caused by paratyphoid fever brought by Europeans. We retrieved genome-wide data from two Spanish soldiers who were besieging the city of Barcelona in 1652, during the Reapers' War. Their ancestry derived from the Basque region and Sardinia, respectively, (at that time, this island belonged to the Spanish kingdom). Despite the proposed plague epidemic, we could not find solid evidence for the presence of the causative plague agent in these individuals. However, we retrieved from one individual a substantial fraction of the *Salmonella enterica* serovar Paratyphi C lineage linked to paratyphoid fever in colonial period Mexico. Our results support a growing body of evidence that Paratyphi C enteric fever was more prevalent in Europe and the Americas in the past than it is today.

## Highlights

• Genome-wide data from two soldiers of the 1652 siege of Barcelona (Spain)
• Retrieval of an ancient *Salmonella enterica* serovar Paratyphi C strain
• Seventeenth century European *S. enterica* Paratyphi C clusters with colonial Mexican strains
• Further support for transatlantic dispersals of paratyphoid fever

## Graphical abstract

**Figure:**
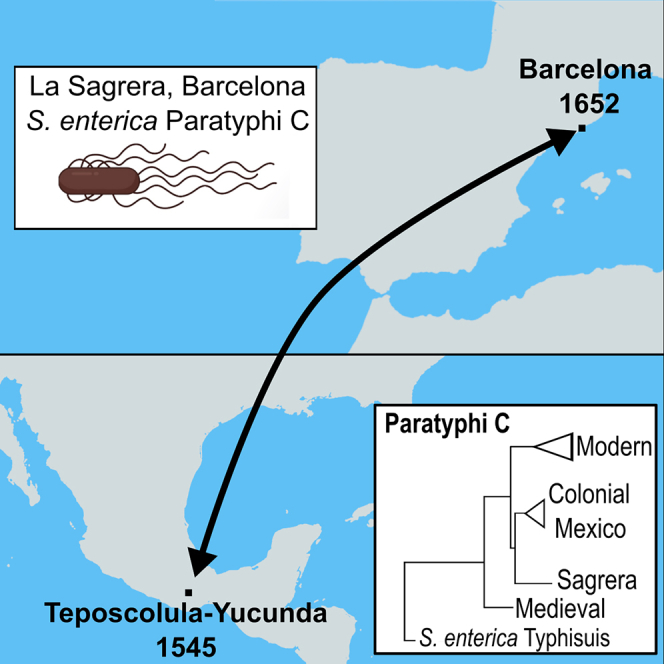

## Introduction

Next generation sequencing techniques allow for the recovery of historic pathogens from a variety of sources including medical collections (De-Dios, 2019; Van Dorp et al., 2020), ancient parchments (de-Dios et al., 2020; Piñar et al., 2020), ancient “chewing gum” (Jensen et al., 2019), and human archaeological remains (Maixner et al., 2016; Mühlemann et al., 2018; Vågene et al., 2018; Zhou et al., 2018; Key et al., 2020). One of the most widely studied historic pathogen is *Yersinia pestis*, the bacterial agent of plague, for which numerous ancient genomes—almost exclusively sequenced from teeth—have been analyzed spanning different time periods and locations (Bos et al., 2011, 2016; Rasmussen et al., 2015; Andrades Valtueña et al., 2017; Rascovan et al., 2019).

More recently, ancient genomics has recovered DNA attributable to the pathogen *Salmonella enterica*, a species comprising over 2500 serovars. These include *Salmonella enterica* serotypes Typhi (or typhoid fever, which accounts for 75% of cases) and Paratyphi A, B, and C (or paratyphoid fevers) (Cash-Goldwasser and Barry, 2018). The former serovar is globally prevalent among paratyphoid fevers (Ochiai et al., 2005; Stanaway et al., 2019), while Paratyphi B and C are relatively scarce (Achtman et al., 2012; Cash-Goldwasser and Barry, 2018). Typhoid and paratyphoid fevers affect up to 14 million people and cause the death of 135,900 people annually (Stanaway et al., 2019). Although a non-life-threatening disease with appropriate antibiotic treatment, without it, mortality rates can reach 10–20% (Crump et al., 2008; World Health Organisation, 2018). Typhoid and paratyphoid fevers are particularly prevalent in developing countries of sub-Saharan Africa, South East Asia, and South Asia, where they represent one of the leading causes of death and disability (Naghavi et al., 2017; Vos et al., 2017).

Genomic analyses of ancient strains of *S. enterica* support its prevalence as a pathogen of humans in the past, with *S. enterica* directly observed in association with humans across Eurasia over at least the last 6500 years (Key et al., 2020). Together with other *Salmonella enterica* serotypes, Paratyphi C belongs to a subgroup of *S. enterica* serovars defined as the Ancient Eurasian Super Branch (AESB), a cluster that comprises phylogenetically close serotypes that infect different wild animals and livestock, as well as strictly human-adapted lineages. The process of host adaptation of the AESB likely accompanied the Neolithic transition, when changes in lifestyle and closer interactions with domesticated animals may have led to recurrent exposure and infections (Key et al., 2020). The characterization of the Paratyphi C genome “Ragna” identified from the sequenced remains of both the bones and the teeth of an 800-year-old skeleton from Trondheim, Norway, demonstrates the presence of Paratyphi C and invasive salmonellosis in past Medieval Europe (Zhou et al., 2018).

More recent evidence for the past epidemic potential of this bacterium is the discovery of Paratyphi C in human burials from colonial Mexico associated with the *Cocoliztli* epidemics (Vågene et al., 2018). The *Cocoliztli* (disease or plague in Nahuatl) was a series of epidemics that devastated the native populations of New Spain after the arrival of Spanish colonizers during the early 16^th^ century (Malvido and Viesca, 1985). These epidemics were believed to have been caused by the introduction of infectious diseases by Europeans with suggested agents including measles, smallpox, malaria, and unknown haemorrhagic fever (Somolinos d’Ardois, 1970; Malvido and Viesca, 1985; Marr and Kiracofe, 2000). Detection of Paratyphi C in association with victims of the *Cocoliztli* lends support to the hypothesis that *S. enterica* was a contributor to epidemics during recent historical times. *S. enterica* has also been suggested as the causative agent of the famous Plague of Athens (430–426 BCE) following the amplification of two DNA fragments from individuals from the ancient *Kerameikos* mass grave, dating to that period (Papagrigorakis et al., 2006). However, subsequent phylogenetic assessment of these sequences could not authenticate them as *S. enterica* (Shapiro et al., 2006). This discrepancy can be partly explained by the difficulties of retrieving significant portions of ancient microbial genomes before the advent of second-generation sequencing technologies. Therefore, although it has been suggested that Paratyphi C was a “globally” distributed pathogen of the past, more evidence based on genome-wide data from different locations and time periods is needed to assess its putative role as an epidemic pathogen.

In this study, we generate and analyze DNA from the teeth of two individuals following archaeological excavations at La Sagrera (Sant Martí de Provençals), the Spanish army encampment that besieged Barcelona (August 1651 to October 1652) during the Reapers’ War (Monguiló et al., 2012). As well as characterizing the ancestry of these two soldiers, we retrieve from one individual a large number of sequence reads mapping to *Salmonella enterica* Paratyphi C. We place the resulting Paratyphi C genome in the context of previously published modern and ancient strains including from medieval Norway and colonial Mexico. The accurate dating of this wartime site, coupled with the observations provided by this genome, extends our understanding of the spatiotemporal breadth of Paratyphi C across Europe and the Americas.

## Results

### Ancient DNA sequencing and characterization of La Sagrera soldiers

Archaeological excavations at La Sagrera uncovered 576 skeletons of soldiers grouped in shallow graves. Most of these contained between one and ten individuals, but they also included two large pits with 69 and 79 individuals, respectively. Based on the archaeological context of the burials, the soldiers were hastily buried—sometimes still dressed and with boots on—without signs of war injuries and within a short period of time. Archaeologists tend to associate such burial procedures with disease outbreaks (Signoli et al., 2004; Raoult et al., 2006). In contemporaneous chronicles, it is mentioned that both the defenders—within the city—and the besiegers suffered a bout of “pestilence” that has been previously considered to be a plague outbreak (Parets, 1660; Luis and Moya, 1990).

**Figure 1:**
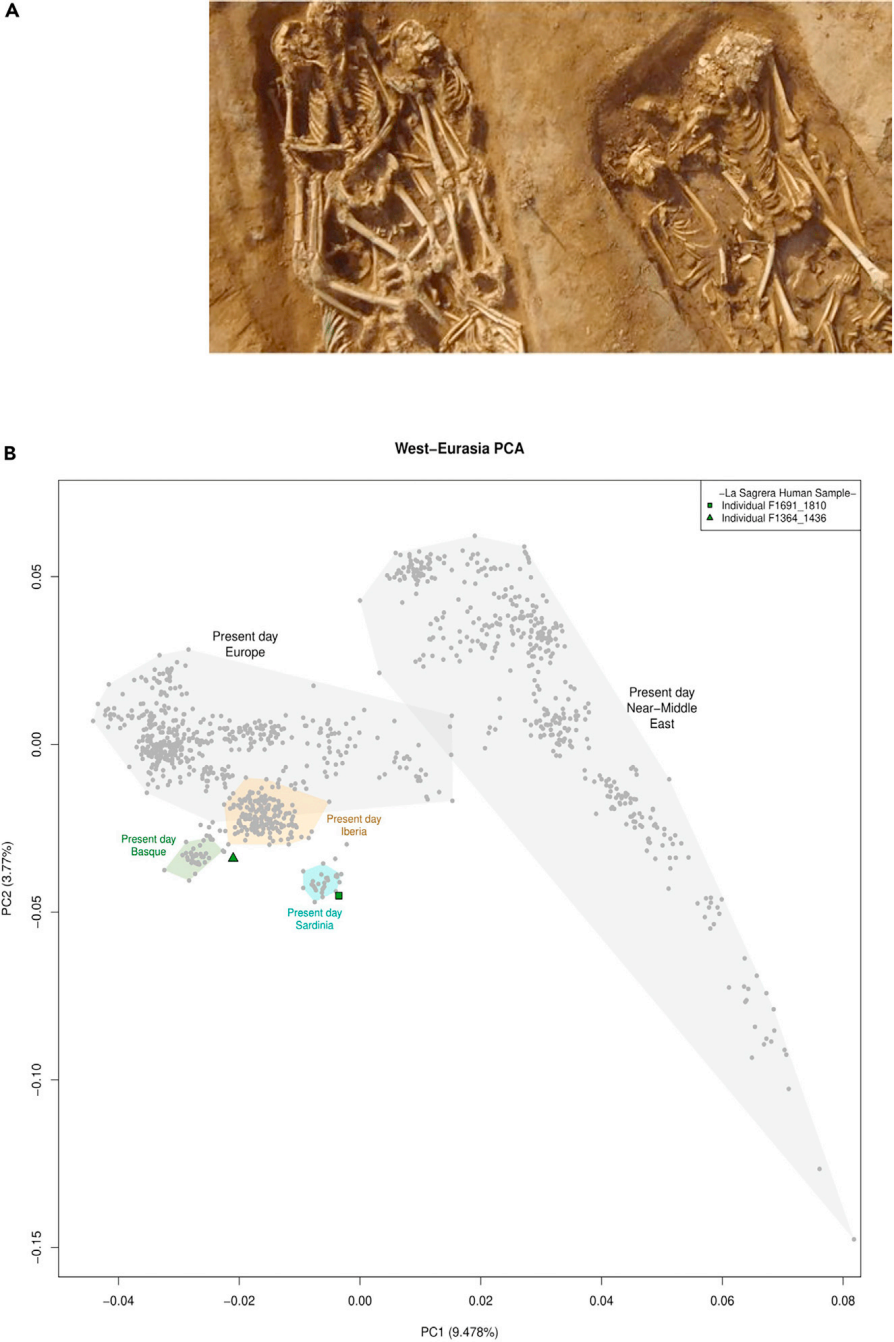
Population genetic affinities of the soldiers analyzed at the archaeological site at La Sagrera (A) Image of some of the soldiers’ remains found at the site. (B) Genetic data for both individuals (see legend at top right) are projected onto the two main principal components (PC) defined by 1431 present-day West-Eurasian individuals genotyped by human origins array (gray-points). Modern populations more closely related to the Siege of Barcelona soldiers are highlighted (present-day Basques (green); Spanish (yellow); Sardinians (cyan)).

We sampled the teeth of two soldiers of the Spanish army, labeled F1691-1810 and F1364-1436, for ancient DNA sequencing (Figure 1A). From these samples, we obtained 246,473,297 and 99,018,404 DNA reads, respectively. The ends of the DNA reads obtained displayed typical ancient DNA (aDNA) damage patterns in a ratio consistent with the estimated age of the site (Figure S1). In both cases, the molecular sex was assigned to male (Table S1). Based on the presence of different mitochondrial DNA (mtDNA) haplogroups and on heterozygosity levels of the X chromosome, we found that one of the libraries was partially contaminated (around 9% of human reads). Therefore, we only considered the non-contaminated libraries for the subsequent human population genetics analysis. The mtDNA haplogroups were assigned as U5b1f1a and H2a5a for F1691-1810 and F1364-1436, respectively (Table S2). Y chromosome haplogroups were assigned with differing degrees of resolution to R1 and R1b1a1b1 for F1691-1810 and F1364-1436, respectively.

### Population genetic affinities of the analyzed individuals

Little is known about the geographic origins of soldiers enlisted in the Spanish army at the time, and uncertainties exist over whether these predominately comprised locally or foreign raised troops. Projection of the diversity detected both individuals into a principal component analysis (PCA) conducted on modern West Eurasians placed F1364-1436 neighboring the diversity observed in present day Basques and F1691-1810 falling close to the genetic diversity observed in present day Sardinians (Figure 1B) (Álvarez-Iglesias et al., 2009; Cardoso et al., 2013). What is now the Basque Country and the island of Sardinia were under dominion of the Spanish crown during the 17^th^ century, implying that all the regions of the Spanish realm contributed with manpower to the war effort. In fact, it is recorded that the fall of Barcelona in 1652 was celebrated across the Spanish possessions, including Cagliari in Sardinia (Caredda, 2015). The analysis of further individuals could provide additional information on the composition of professional Spanish armies during the 17^th^ century; for example, contemporaneous chronicles explain there was a contingent of Irish mercenaries (Morales Ó, 2004).

### Ancient pathogens screening of La Sagrera soldiers

**Figure 2:**
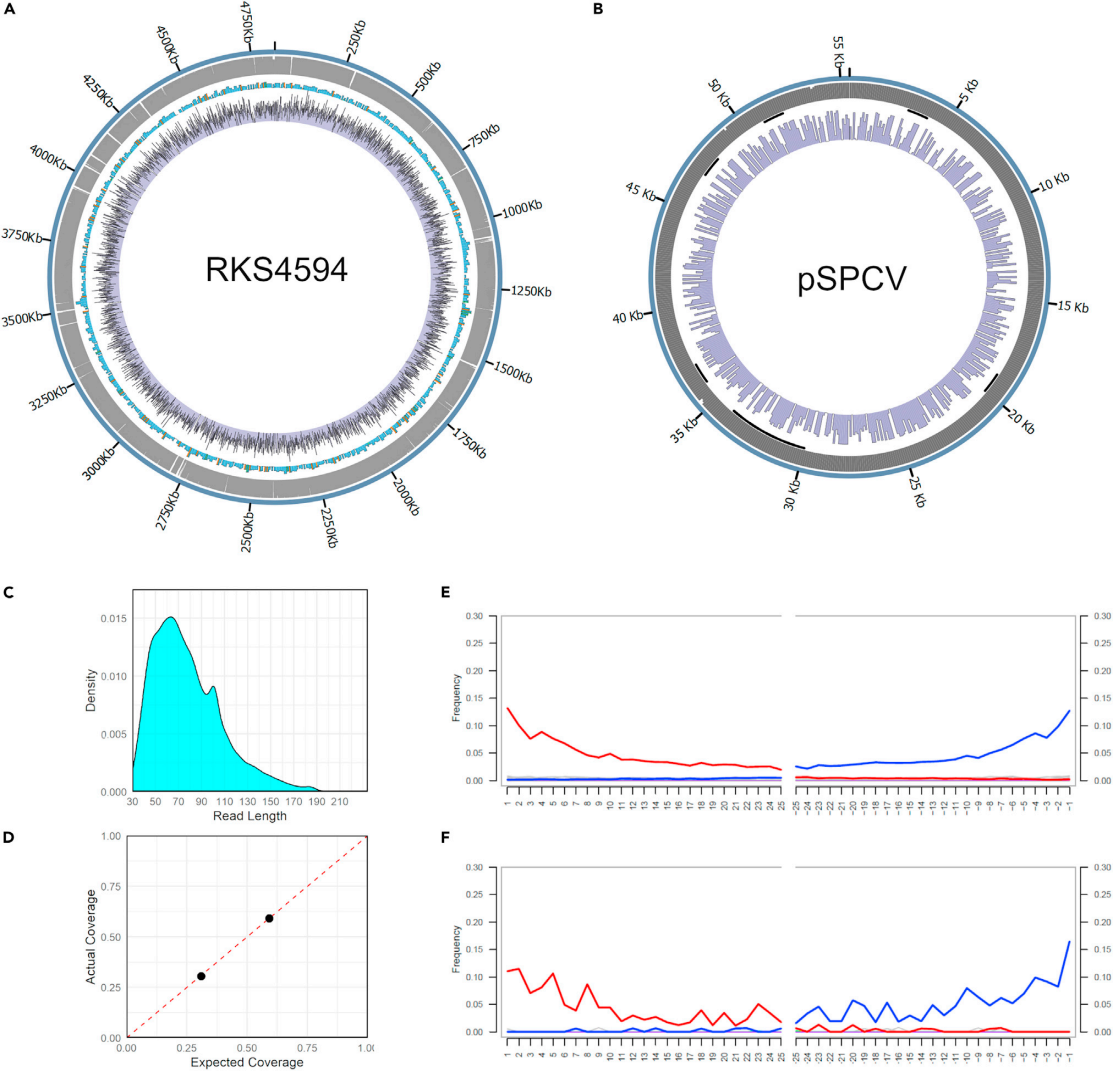
Characteristics of the *S. enterica* Paratyphi C recovered sequences (A and B) Coverage plot of the Paratyphi C chromosome (A) and plasmid (B); the outer blue circle provides the genomic position, the second outermost circle in gray provides the reference mappability, the third most outermost ring provides the presence of genes (blue), pseudogenes (orange), and RNAs (green) along the reference, and the innermost purple circle provides the mean depth (binned) of coverage. (C) Read length distribution of sequences mapped against Paratyphi C reference genome. (D) Comparison between the calculated and expected coverage and the actual observed coverage for both the pSPCV plasmid and chromosome. (E) aDNA damage patterns (site specific substitutions) at both ends of the DNA reads mapped against the Paratyphi C reference genome. (F) damage patterns at both ends of the DNA reads mapped to the pSPCV plasmid. For both (E) and (F), red provides the C to T substitution frequency; blue provides the G to A substitution frequency; and gray provides the frequency relative to all other substitutions.

Although contemporaneous assessments suggested that soldiers died of plague during the siege (Luis and Moya, 1990), metagenomic screening of the non-human DNA content from both individuals was unable to identify the presence of *Yersinia pestis* DNA reads; only 0.00003%–0.000003% of all DNA reads were assigned to pathogenic *Yersinia* species (Table S3). The absence of *Y. pestis* in these individuals does not exclude outbreaks of plague during the siege, noting that identification of *Y. pestis* tends to require large-scale screening of individuals. Interestingly, 0.01% of sequencing reads were assigned to *Salmonella* in one of the individuals F1691-1810, with only 0.00001% in the other. After mapping all reads in F1691-1810 against a comprehensive set of *Salmonella enterica* reference genomes, *Paratyphi C* was determined as the best representative based on the mean coverage, the percentage of the genome covered by at least a single read, and the mean read edit distance (Table S4) (Liu et al., 2009). Mapping solely against the *Paratyphi C* reference chromosome (see STAR Methods), we obtained 24,225 uniquely mapped DNA reads, accounting for 30% of the reference genome at an average depth of coverage of 0.38X (Table S5). In addition, we obtained 59.02% and an average depth of coverage of 0.91X over the ∼5 4kb Paratyphi C virulence plasmid (pSPCV), supporting its vertical inheritance and co-evolution with the Paratyphi C lineage (Zhou et al., 2018; Key et al., 2020). The expected coverage for both the chromosome and the plasmid matched the expected theoretical value (Figures 2A, 2B and 2D) (see STAR Methods). Postmortem damage at the end of the *Salmonella* DNA reads (up to 15%) and read length distribution, as well as their absence in two extraction blanks (Table S5), supported these sequences as authentically old rather than modern contaminants (Figures 2C, 2E, and 2F).

### Phylogenetic analysis of the *Salmonella enterica* Paratyphi C-retrieved sequences

After filtering out sequences by depth, heterozygosity, and the presence of low coverage transitions (see STAR Methods), we generated 1,146,808 confidently covered positions, of which 10,250 are single-nucleotide polymorphisms (SNPs). Finally, we explored the depth of coverage in virulence-related regions associated with the pSPCV (NC_012124.1) plasmid (Liu et al., 2009) and other virulence-associated genes. No apparent variation in coverage over these genes was detected, suggesting that virulence gene composition of pSPCV has been largely maintained from the time of our sample to the present day (Figure S2; Table S6).

To place our strain into the wider context of *Salmonella enterica*, we built a maximum likelihood (ML) tree over the chromosomal alignment that included 415 isolates (both ancient and modern), encompassing the AESB clade and including *S. enterica* serovar Enteritidis as an outgroup. This placed our 17th-century Barcelona strain within the diversity of sampled Paratyphi C. Repeating the ML tree using only Paratyphi C strains and Typhisuis as an outgroup, F1691-1810 was positioned in a clade (with 100% bootstrap support) falling basal to all modern Paratyphi C diversity and including the colonial Mexican Paratyphi C strains associated with the *Cocoliztli* epidemics (Figures S3A and S4) (Vågene et al., 2018). The only strain basal to this clade is the medieval sample from Trondheim (Norway) dated to 1,200 CE (Zhou et al., 2018). Under the expectation of constant accumulation of mutations with time, the terminal branch lengths of the ancient samples are longer than seen in modern strains, suggesting the possibility of recombination events or downstream incorporation of SNPs which may be erroneously called due to low coverage or postmortem damage (Figure S3B).

Due to the high genetic affinity and close temporal range of the Paratyphi C genome obtained from the F1691-1810 La Sagrera individual and the ancient lineage associated with the Mexican *Cocoliztli*, we explored if the split time of these clades was more consistent with an introduction of *S. enterica* Paratyphi C into the Americas by Spanish colonizers or if the strain from Barcelona was imported into Spain from the American continent. To create a robust alignment suitable for the application of phylogenetic tip dating, we first set out to prune from the alignment all sites in conflict with the expectation under clonal evolution. In particular, we applied *ClonalFrameML* (Didelot et al., 2015) to identify all putative homologous recombination events between sets of donors and recipients in the Paratyphi C phylogeny. We identified putative recombinant tracts comprising a total of 104 different events totaling 30 kb in length (Figures S5 and S6; Table S7).

### Temporal relationship of La Sagrera with other taxa in the Paratyphi C clade

**Figure 3:**
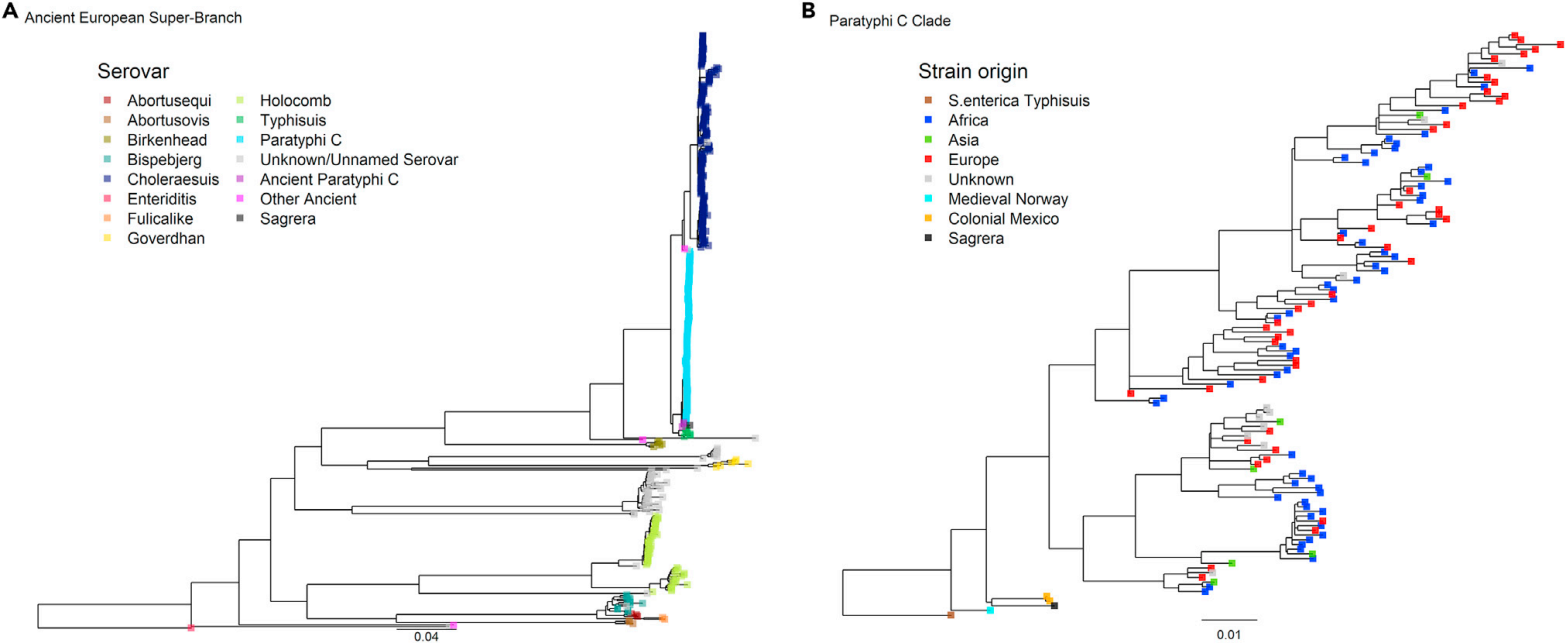
Maximum likelihood phylogeny of *S. enterica* falling within the Ancient Eurasian Super Branch (AESB). (A) ML tree of La Sagrera and 413 *S. enterica* strains representing the ancient and modern diversity of the AESB from previous studies (Vågene et al., 2018; Zhou et al., 2018; Key et al., 2020). The tree is rooted using *S. enterica* ser. Enteriditis from a previous study (Alikhan et al., 2018). Serovars are colored at the branch tip. (B) Maximum likelihood phylogeny of the Paratyphi C clade including 124 Paratyphi C modern and ancient strains. The tree includes La Sagrera strain, 119 Modern Paratyphi C strains (Key et al., 2020), 2 strains from Colonial Mexico (Vågene et al., 2018), a strain from Medieval Norway (Zhou et al., 2018), and an *S. enterica* ser. Typhisuis as an outgroup (Key et al., 2020). Tip points are colored according to their origin as given by the legends. In both cases, phylogenies were constructed from clonal alignments with recombination events and homoplasies removed.

In addition, we applied a further strict filtering technique, identifying all homoplastic positions in the alignment given the consensus relationships supported by the ML phylogeny. Such sites may derive from recombination events otherwise undetected by *ClonalFrameML* or correspond to spurious variant calls included as a result of low coverage or DNA damage. Applying *HomoplasyFinder* (Crispell et al., 2019), we identified homoplasies by calculating the consistency index, which, for each site, provides a measure of the observed number of changes divided by the minimum number of changes needed to achieve a certain state at tip of a tree. We detected 532 positions in the alignment with a consistency index <0.5 and subsequently pruned these from the recombination-stripped Paratyphi C alignment (Crispell et al., 2019). The resulting filtered alignment comprised 3680 variant sites fully covered across all of the 124 Paratyphi C strains. We generated a further ML phylogeny using only those positions that passed the filtering criteria. The new tree was topologically congruent; however, the terminal branch length of the ancient tips in the tree was reduced, suggesting that the alignment filtering procedure removed recombinant and/or spurious sites (Figure 3).

**Figure 4:**
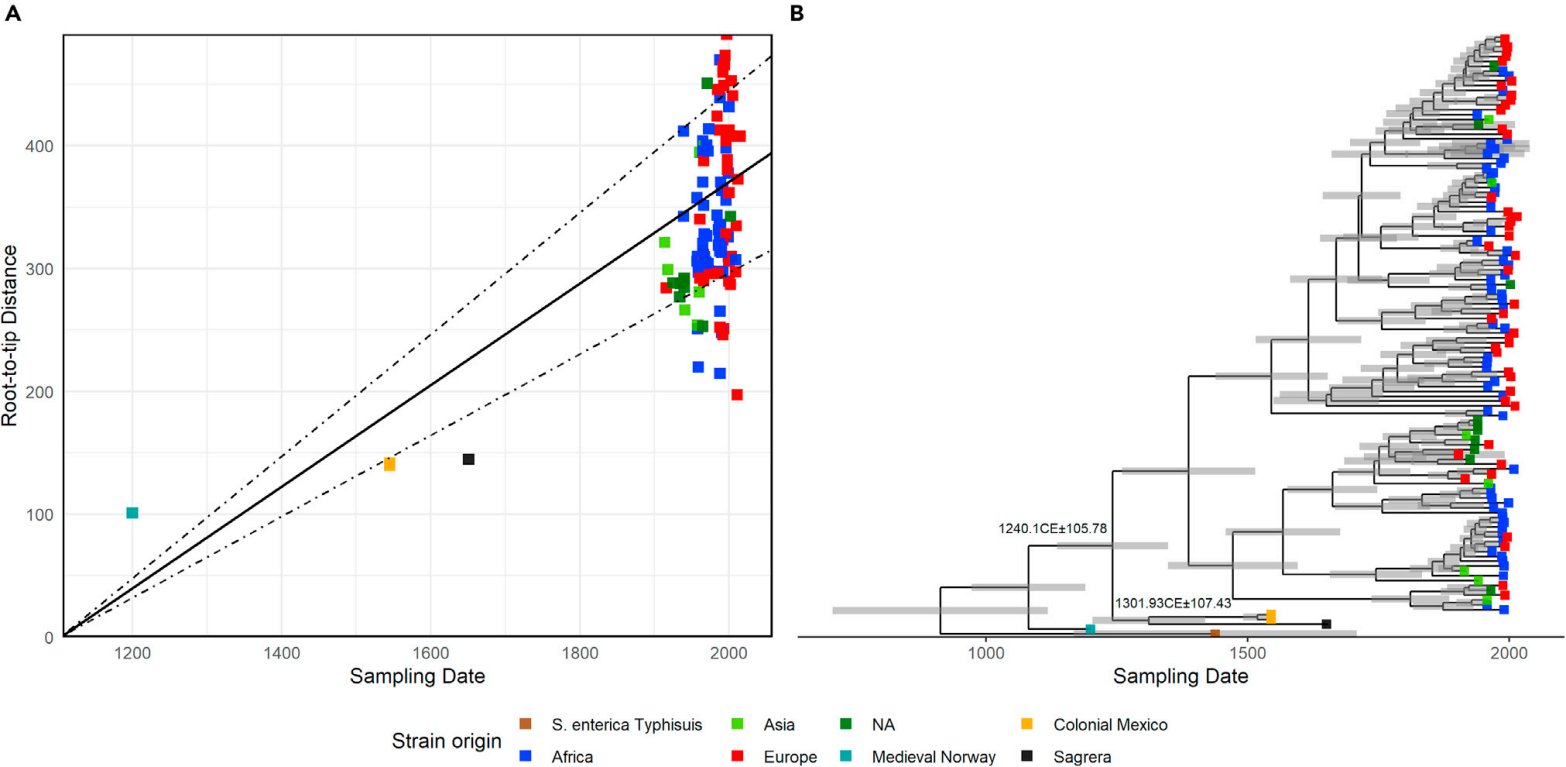
Temporal signal in the Paratyphi C clade (A) Regression of the root-to-tip distance and collection date of the samples present in the tree, with 1 × 10^7^ permutations. R^2^ = 0.44, p < 1.00 × 10^−4^. Dotted lines provide the 95% CI. (B) Time-calibrated phylogenetic tree of the curated Paratyphi C dataset. X axis provides the time of collection date. Gray bars at each node denote the 95% CI of the estimated divergence times. Key divergence estimates are highlighted and discussed in the text.

Previous studies have utilized ancient Paratyphi C genomes to calibrate the evolutionary rate for members of the AESB (Zhou et al., 2018; Key et al., 2020). Similarly, we estimate the age of the most recent common ancestor (MRCA) (the split times) of our sample at relevant nodes by calibrating the recombination-filtered phylogenetic tree by time. To do so, we first confirmed the existence of a significant temporal signal over the alignment by computing the correlation between the root-to-tip genetic distance and the estimated time of sample collection. For our ancient Spanish sample, we set a prior on the tip date to 1652 to coincide with the timing of the siege of Barcelona and the archaeological dating of the site. We recovered a positive correlation (R^2^ = 0.3) between the root-to-tip distance and sampling time, which was significant following 1 × 10^4^ randomizations of the tip sampling date (p < 1.00 × 10^−4^) (Figure 4A). We then applied *BactDating* to formally estimate the mutation rate over the alignment running until full convergence of the MCMC algorithm (Didelot et al., 2018) (Figure S7). We estimated a mutation rate for this clade over the clonal frame to 1.02 × 10^−4^ mutations per site per year. This mutation rate corresponds well with the rates reported for other *S. enterica* serovars (Okoro et al., 2012; Hawkey et al., 2013; Octavia et al., 2015) and is similar to recent estimates following equivalent approximations (Didelot et al., 2021).

Based on the application of an additive clock model, the estimated rate of evolution leads to an inferred date of the split between the genome identified in medieval Trondheim (Ragna) and the rest of the diversity of Paratyphi C to 1058.43 CE (CI: 949.85 CE–1167.0 CE), a timing consistent with an equivalent analysis by Zhou et al. (2018) (1162–1526 ybp, CI: 833–1975 ybp), thereby supporting the robustness of temporal estimates when including our low coverage sample. We estimate the divergence time between the 1652 La Sagrera Spanish genome and samples from colonial Mexico from sampled modern diversity to 1240.11 CE (CI: 1134.32 CE–1345.89 CE). Finally, we estimated the split between our 1652 sample and those linked solely to New World epidemics in Mexico to 1301.93 CE (CI: 1194.5 CE–1409.35 CE) (Figure 4B), suggesting a common ancestor of Mexican and the Spanish Paratyphi C around the turn of the 14^th^ century.

## Discussion

Despite an expectation that the two soldiers whose remains we analyzed had died of the plague (Parets, 1660; Luis and Moya, 1990), no reliable traces of *Yersinia pestis* could be found. This does not preclude plague outbreaks during the Siege of Barcelona; a far larger number of skeletons will have to be screened before *Y. pestis* can be formally ruled out as the agent of the disease that affected Barcelona’s besiegers. However, we did recover the partial genome of the pathogen *Salmonella enterica* Paratyphi C in one soldier, which may have contributed to his demise, possibly as part of a wider epidemic affecting the Spanish army. Indeed, outbreaks of enteric fever are plausible considering the sanitary conditions of a cramped military camp during a siege, as well as the surrounding marshes (that were drained in modern times (Martín-Vide, 2015)). Both of these factors and the presence of contaminated water may favor the rapid transmission of disease (Gunn et al., 2014). Nonetheless, it is not possible to rule out the co-existence of *Y. pestis* given that so far we have only analyzed two individuals and the fact that Plague was a well-known disease at the time with clear symptoms (Parets, 1660; Luis and Moya, 1990).

Recent studies have demonstrated the possible epidemic character of *S. enterica* Paratyphi C in historical times. For example, *Salmonella* Paratyphi C DNA was found in ancient remains from the New World (Vågene et al., 2018) associated with mass burials in Mexico dating back to the mid-16^th^ century, plausibly attributed to the *Cocoliztli* epidemics. The discovery and characterization of the pathogen in a 12^th^ century woman from Trondheim, Norway, further supports the notion that *S.* Paratyphi C may have noticeably contributed to past pandemics of enteric fever in recent human history (Zhou et al., 2018), despite being rare in Europe and the Americas today.

Our work, recovering a ∼1652 *S. enterica* Paratyphi C genome from a Spanish soldier, extends the spatiotemporal distribution of *S.* Paratyphi C to Southern Europe to as recently as the mid-17^th^ century and provides a further observation of the presence of this pathogen in epidemic burial context. The phylogenetic placement of our Paratyphi C genome from 1652 Barcelona basal to the diversity observed in post-contact Mexican strains supports one of two scenarios: (i) an introduction of S. Paratyphi C strains from Europe or (ii) an extensive geographic range of S. Paratyphi C prior to the colonial period. Given that other cases of introduction of pathogens in the Americas during the colonial period have been recently proposed, aided by the analysis of ancient microbial genomics, including parvovirus and hepatitis B virus, leprosy, syphilis, and malaria (Yalcindag et al., 2012; Schuenemann et al., 2013; Guzmán-Solís et al., 2020; Majander et al., 2020; Van Dorp et al., 2020), it is plausible that colonial migrations contributed to the distribution of past Paratyphi C observed. The continental origin of some infectious diseases putatively associated with colonial settlement, such as syphilis, has been debated for decades with little progress from the analysis of modern strains alone. Syphilis was once thought to have been introduced in Europe from the Americas; however, strains likely predating Columbian times have been recently discovered in Northern European skeletal remains (Schuenemann et al., 2018; Majander et al., 2020).

The presence of *S.* Paratyphi C in mid-17th century Spain is particularly interesting in the context of these historical epidemics due to the fact that these strains are no longer present in the current world diversity of *S.* Paratyphi C. Our estimated split between the Spanish and Mexican strains to the early 14^th^ century predates Columbian times. Such an observation may be consistent with a possible European reservoir in Spain of the *Salmonella* lineages which are postulated to have caused widespread epidemics in the native populations of the Americas during the discovery epoch. Adding support to our estimates, the divergence dates we recover over the wider phylogeny, for instance, the split time of *S. enterica* Paratyphi C from *S. enterica* Typhisuis, are highly consistent with independent estimates obtained in studies providing the first characterizations of the timing of evolutionary events in this serovar using ancient DNA (Zhou et al., 2018; Key et al., 2020). This suggests the inclusion of even partial ancient genomes, as we obtained here, can be useful in temporal phylogenomic analyses. Despite uncertainties associated to phylogenetic tip-dating methods and to the low coverage data from ancient skeletal remains, our results reinforce the presence of *S. enterica* Paratyphi C in Europe for centuries, opening the possibility that this pathogen contributed to other historical pandemics of debated cause—including the one suffered by Spanish besiegers of Barcelona in 1652.

Nevertheless, the likely complex pattern of trans-Atlantic connectivity detected here may only be fully clarified with the study of additional ancient samples, both from the Americas, Africa, and Eurasia. One challenge caused by the lack of osteological signals associated with typhoid fevers means that such a survey will need to be conducted blindly, analyzing, like we did in this study, historical mass graves potentially attributed to other common past pandemics such as the plague.

### Limitations of the study

It has not been possible to retrieve the full *S. enterica* Paratyphi C genome, and the low coverage may have resulted in the inclusion of erroneous sites despite our stringent filters (Figure S8). It is possible this could subtly impact some of our temporal estimates. In the future, we would like to generate DNA from remains of additional soldiers from this site in the hope to identify further, higher coverage, representatives of the clade.

## STAR★ Methods

### Key resources table

**Table:** 

REAGENT or RESOURCE	SOURCE	IDENTIFIER
**Biological Samples**		

Tooth from a 17^th^ century soldier	La Sagrera site (Barcelona, Spain)	F1364-1436
Tooth from a 17^th^ century soldier	La Sagrera site (Barcelona, Spain)	F1691-1810

**Deposited Data**		

Sequencing data	This paper	ENA Project: PRJEB43276
Human reference genome NCBI build 37, GRCh37	Genome Reference Consortium	http://www.ncbi.nlm.nih.gov/projects/genome/assembly/grc/human/
Human Origins Dataset	(Lazaridis et al., 2016)	https://reich.hms.harvard.edu/datasets
Y chromosome haplogroup database version 15.73	International Society of Genetic Genealogy	http://www.isogg.org
*Yersinia enterocolitica* assembly ASM25317v1	(Batzilla et al., 2011)	https://www.ncbi.nlm.nih.gov/assembly/GCF_000253175.1/
*Yersinia pestis* assembly ASM379822v1	Food and Drug Administration	https://www.ncbi.nlm.nih.gov/assembly/GCF_003798225.1/
*Yersinia pseudotuberculosis* assembly 51108_B01	Wellcome Sanger Institute	https://www.ncbi.nlm.nih.gov/assembly/GCF_900637475.1/
*Yersinia similis* assembly ASM58251v1	(Sprague and Neubauer, 2014)	https://www.ncbi.nlm.nih.gov/assembly/GCF_000582515.1/
*Salmonella enterica* serovar Paratyphi C assembly RKS4594	(Liu et al., 2009)	https://www.ncbi.nlm.nih.gov/assembly/GCF_000018385.1/
Plasmid Database	(Acman et al., 2020)	https://github.com/macman123/plasmid_network_analysis
*Salmonella enterica* AESB strains whole genome data	(Key et al., 2020)	http://enterobase.warwick.ac.uk/species/senterica/search_strains?query=workspace:12971
*Salmonella enterica* Enteriditis whole genome data	(Alikhan et al., 2018)	NCBI Sample: ERS217420
Medieval Norway *Salmonella enterica* Paratyphi C whole genome data	(Zhou et al., 2018)	ENA Project: PRJEB19916
Colonial Mexico *Salmonella enterica* Paratypic C whole genome data	(Vågene et al., 2018)	ENA Project: PRJEB23438
Phylogenetic tree of “la Sagrera” *Salmonella* sequences within the Paratyphi C diversity	This Paper	https://microreact.org/project/G7XdKYogeqhqkWBQLu34t
Recombination tracks of “la Sagrera “– Paratyphi C phylogeny	This Paper	N/A
Dation of “la Sagrera “– Paratyphi C phylogeny	This Paper	N/A

**Software and algorithms**		

AdapterRemoval v.2.2.2	(Schubert et al., 2016)	https://github.com/MikkelSchubert/adapterremoval
BWA v.0.1.17	(Li and Durbin, 2009)	http://bio-bwa.sourceforge.net/
SAMtools v.1.12	(Li et al., 2009)	http://www.htslib.org/
picard v.2.25.5	(Broad Institute, 2018)	https://broadinstitute.github.io/picard/
Qualimap v.2.2	(Okonechnikov et al., 2016)	http://qualimap.conesalab.org/
pmdtools v.0.60	(Skoglund et al., 2014)	https://github.com/pontussk/PMDtools
mapDamage2 v.2.0.8	(Jónsson et al., 2013)	https://ginolhac.github.io/mapDamage/
Ry_compute v.0.4	(Skoglund et al., 2013)	https://github.com/pontussk/ry_compute/blob/master/ry_compute.py
Haplogrep2 v.2.1.21	(Weissensteiner et al., 2016)	https://haplogrep.i-med.ac.at/app/index.html
schmutzi v.1.5.4	(Renaud et al., 2015)	https://github.com/grenaud/schmutzi/tree/master
angsd v.0.925	(Korneliussen et al., 2014)	http://www.popgen.dk/angsd
EIGENSOFT v.7.2.1	(Patterson et al., 2006; Price et al., 2006)	https://www.hsph.harvard.edu/alkes-price/software/
prinseq v.0.20.4	(Schmieder and Edwards, 2011)	https://sourceforge.net/projects/prinseq/files/
Kraken2 v.2.1.1	(Wood et al., 2019)	http://www.ccb.jhu.edu/software/kraken2/
bedtools v.2.24.0	(Quinlan and Hall, 2010)	https://bedtools.readthedocs.io/en/latest/
GATK v.3.7	(Van der Auwera et al., 2013)	https://gatk.broadinstitute.org/hc/en-us
BamUtil v.1.0.13	(Jun et al., 2015)	https://github.com/statgen/bamUtil
RAxML v.8.2.4	(Stamatakis, 2014)	https://cme.h-its.org/exelixis/web/software/raxml/
R v.4.1.0	(Ihaka and Gentleman, 1996)	https://cran.r-project.org/
Ggtree v.1.14.6	(Yu et al., 2017)	https://guangchuangyu.github.io/software/ggtree/
iTOL v.6	(Letunic and Bork, 2019)	https://itol.embl.de/
ClonalFrameML v.1.2	(Didelot et al., 2015)	https://github.com/xavierdidelot/ClonalFrameML
HomoplasyFinder v. 0.0.0.9	(Crispell et al., 2019)	https://github.com/JosephCrispell/homoplasyFinder
BactDating v.1.0.11	(Didelot et al., 2018)	https://github.com/xavierdidelot/BactDating
ggplot2 v.3.0.0	(Wickham, 2016)	https://cran.r-project.org/web/packages/ggplot2/index.html
Ape v.5.4	(Paradis and Schliep, 2019)	https://cran.r-project.org/web/packages/ape/index.html
CODA v.0.19-4	(Plummer et al., 2006)	https://cran.r-project.org/web/packages/coda/index.html

### Resource availability

#### Lead contact

Further information and requests for resources and reagents should be directed to and will be fulfilled by the lead contact, Carles Lalueza-Fox carles.lalueza.fox@gmail.com.

#### Materials availability

This study did not generate new unique reagents.

### Experimental model and subjects details

#### The site

A recently excavated archaeological site located in La Sagrera -currently a north-eastern quarter of the city of Barcelona- dates from 1651-1652 CE, during the conflict known as the Reapers’ War (*Guerra dels Segadors,* 1640-1659), the last stage of the Thirty Years’ War (Monguiló et al., 2012). During 1651, the city was under siege by Spanish forces commanded by Juan José de Austria. Despite the efforts of the local garrison and the arrival of French reinforcements, the city capitulated in the spring of 1652. The site consists of multiple mass graves containing mostly male individuals (118 out of 140 studied by physical anthropologists so far), with ages ranging from 16 to 40 years and skeletal hallmarks of having undertaken intense physical activities. The individuals analyzed in this study come from the burial F1691 (individual 1810) and F1364 (individual 1436). An upper canine was extracted from each of the skulls and used for genetic analyses. The chronology was confirmed by a direct radiocarbon dating of one individual (F1334) that yielded a date of 360+/-30 years BP (Beta-573178) and by the analysis of 152 coins found across the site. Of these, 104 coins correspond to coinage minted in the first half of the 17th century from Philip IV of Spain and also Louis XIII and XIV of France. The remaining 48 coins are a diverse assembly of coins minted during the second half of the 16th century and the first half of the 17th century.

### Method details

#### DNA extraction and library preparation

All DNA extraction and initial library preparation steps (prior to amplification) were performed in a dedicated ancient DNA laboratory, physically isolated from the laboratory used for post-PCR analyses. Strict protocols were followed to minimize the amount of human DNA in the ancient DNA laboratory, including the existence of positive air pressure in the clean rooms, the wearing of a full body suit, sleeves, shoe covers, clean shoes, facemask, hair net and double gloving. All lab surfaces, consumables, disposables, tools and instruments were wiped with bleach and ethanol, and UV irradiated before and after use. First, the teeth samples were UV irradiated (245 nm) for 10 minutes and the outermost surface of the teeth was scrapped off with a drill engraving cutter, followed by another UV irradiation for 10 more minutes, in order to exclude surface DNA contamination. Second, approximately 30 mg of tooth cementum were drilled into a fine powder by a Dremel drilling machine at low speed (5000 rpm). The fact that despite these precautions one initial library was contaminated suggests this took place at the excavation itself, probably during handling. Such an observation would suggest it is advisable to excavate, even recent sites such as this one, with anti-contamination protocols such as those implemented in far more ancient samples (Fortea et al., 2008).

DNA extraction from tooth powder was performed following the method proposed by Dabney et al. (2013). The teeth-powder samples, including an extraction blank, were added to 1ml of extraction buffer (final concentrations: 0.45 M EDTA, 0.25 mg/mL Proteinase K, pH 8.0), resuspended by vortexing and incubated at 37°C overnight (24h) on rotation (750-900 rpm). Remaining tooth powder was then pelleted by centrifugation in a bench-top centrifuge for 2 min at maximum speed (16,100 × g). The supernatant was added to 10mL of binding buffer (final concentrations: 5 M guanidine hydrochloride, 40% (vol/vol) isopropanol, 0.05% Tween-20, and 90mM sodium acetate (pH 5.2)) and purified on a High Pure Extender column (Roche). DNA samples were eluted with 45μl of low EDTA TE buffer (pH 8.0).

A total of 35μl of each DNA extract were converted into Illumina sequencing libraries following the BEST protocol (Carøe et al., 2018). Each library was amplified by PCR using two uniquely barcoded primers. After index PCR, libraries were purified with a 1.5x AMPure clean (Beckman Coulter) and eluted in 25μl of low EDTA TE buffer (pH 8.0). Libraries were quantified using BioAnalyzer and sequenced by HiSeq 4000 (Illumina).

#### Human DNA mapping

Reads were trimmed of sequencing adapters, filtered for reads of less than 30bp and merged using *AdapterRemoval* with default parameters (Schubert et al., 2016). Clipped reads were then mapped against the human reference genome hg37/19 and the Revised Cambridge mitochondrial DNA reference sequence using *BWA aln/samse* with the seeding option disabled (Andrews et al., 1999; Li and Durbin, 2009; Li et al., 2009; Church et al., 2011; Schubert et al., 2012). Next, duplicated reads were removed using *Picard* and only reads with a mapping quality equal or above 30 were considered for downstream analysis (Broad Institute, n.d.). Mapping statistics were calculated using *SAMtools* and *Qualimap2* (Li et al., 2009; Okonechnikov et al., 2016). Finally, to assess the ancient authenticity of the DNA reads, *post-mortem* associated DNA damage was estimated using *mapDamage2* and *PMDtools* (Jónsson et al., 2013; Skoglund et al., 2014).

#### Sex determination and uniparental markers analysis

Molecular sex was assigned using *Ry_compute*, a script designed to determine the sex of individuals sequenced at low coverage based on the ratio of reads mapping to each sex chromosome (Skoglund et al., 2013). Mitochondrial haplogroups were determined using haplogrep2 (Weissensteiner et al., 2016). Y chromosome haplogroup determination was performed by manually annotating variants from the International Society of Genetic Genealogy (http://www.isogg.org).

#### Contamination estimates

Modern human contamination was estimated using two approaches. For mitochondrial contamination, we applied *Schmutzi,* which calculates the degree of modern contamination by considering the profile of aDNA associated deamination in the sample (Renaud et al., 2015). For assessing nuclear DNA contamination, and considering that both individuals are compatible with being males, we estimated the exogenous DNA contamination based on the heterozygosity of the X chromosome sites using *angsd* (Korneliussen et al., 2014) To discard contamination in the reagents, two extraction blanks generated along the project were also sequenced.

#### Human population genetics analysis

To analyze these 17th-century individuals in the context of present-day human genetic diversity, their genomic data was merged with 1134 West-Eurasian individuals genotyped in the Human origins (HO) array (Patterson et al., 2012; Lazaridis et al., 2014, 2016; Biagini et al., 2019). Principal Component Analysis (PCA) was computed using the modern HO individuals, and the ancient samples were projected onto the first two components (PC1 and PC2) using options ‘lsqproject: YES’ and ‘shrinkmode: YES’ of *smartpca* built-in module of EIGENSOFT (Patterson et al., 2006; Price et al., 2006).

#### Pathogens' screening and Salmonella sequences mapping

To explore the presence of relevant microbial organisms in the samples we collapsed unique reads from the human-free sequences and removed from the dataset low complexity sequences using *Prinseq* (Schmieder and Edwards, 2011). Afterward, we applied *kraken2* to assign reads against a standard database (bacteria, archaea, fungi, protozoa and viral) (Wood et al., 2019). We found almost no evidence of *Yersinia pestis* DNA reads, but one of the samples indicated a significant presence of *Salmonella enterica*.

To validate this signal, and to identify the closest modern representative to our sample amongst a diverse set of *S. enterica*, we downloaded 839 *Salmonella* published assemblies from NCBI (as for 03/04/2020) and created a custom database. Human-free reads were then mapped against this database using the local alignment algorithm *mem* of BWA (Li and Durbin, 2009). We mapped the sequences resultant of the local alignment of the free DNA reads against each *Salmonella* assembly downloaded independently using BWA’s global alignment algorithm *backtrack*. The settings used were then optimized for mapping ancient genomes by disabling the seeding option, setting an edit distance value of 0.01 and a gap open penalty value of 2. After that, duplicated sequenced were removed using *Picard* tools and DNA reads with mapping quality >25 were retained (Schubert et al., 2012). Likewise, the absence of *Y. pestis* sequences in the samples was checked by mapping against the *Y. pestis, Y. pseudotuberculosis, Y. similis* and *Y. enterocolitica* reference genomes, and afterward, possible candidate sequences were explored for taxonomic origin using blast to further asses their authenticity (Altschul et al., 1990). The mapping statistics and presence of *post-mortem* aDNA damage were determined as described for the human sequences.

We determined that the most suitable assembly to map against was the *Salmonella enterica subsp. enterica* ser. Paratyphi C reference genome (Liu et al., 2009) (NCBI accession GCF_000018385.1). The number of mapped reads, the mean coverage, the fraction of the genome recovered, and the mean edit distance are provided in Table S4. The presence of *Salmonella* related virulence plasmids was searched by mapping the raw sequences against a comprehensive plasmid database (Acman et al., 2020).

#### Coverage and mappability of Salmonella enterica Paratyphi C

We determined the mappability of the reads to the Paratyphi C reference genome by mapping *k-mers* of length 40 to 100 base pairs. The coverage of the sample was determined using *bedtools* specifying windows of 1kb for the chromosome and 100 bases for the plasmid (Quinlan and Hall, 2010). To ascertain if the fraction of the reference recovered with our sample matches the expected coverage given the sequences’ characteristics we assumed a random distribution matching the actual coverage, assuming sample authenticity, following a previously described approximation (Rasmussen et al., 2015) which calculates the probability of a position being covered given the presence of *r* reads of length *l* using the following formula: $c=1-\overset{N}{\underset{i=1}{\prod }}{\left(1-l_{i}/g\right)}^{r_{i}}$, in which *N* are the different *l*_*1*_ to *l*_*N*_, read lengths with counts *r*_*1*_ *to r*_*N*_. This value must be corrected by multiplying *c* for the mappability of the reference genome, otherwise the true coverage may not match the expected. An additional scenario, leading to the expected and actual coverage not matching, is when the reference has a region with no coverage in the ancient sample (Rasmussen et al., 2015).

#### Virulence gene presence

The sequences of a set of previously described virulence-related genes in Paratyphi C were concatenated to create a reference panel (Connor et al., 2016; Vågene et al., 2018). We mapped our sequences against this reference using the parameters described in the previous section. The percentage of recovered positions for each gene was calculated using *bedtools* and visualized using *ggplot2* (Quinlan and Hall, 2010; Wickham, 2016).

#### Variant calling and phylogenetics dataset creation

Additionally, a dataset of 407 modern *Salmonella enterica* representative of the *AESB* (Alikhan et al., 2018; Key et al., 2020; Zhou et al., 2020), as well as five historical samples (Vågene et al., 2018; Zhou et al., 2018; Key et al., 2020) were curated. For all modern samples raw read data processing was as described for F1691-1810 except for employing the *BWA mem* algorithm with default settings instead of the algorithm *backtrack*, the latter being advised for ancient DNA mapping pipelines. Historical samples were processed as described for the ancient samples. Variants were called from processed published sequences using *GATK* algorithm *UnifiedGenotyper* (Depristo et al., 2011; Van der Auwera et al., 2013). For each sample, positions were filtered for minimum coverage of 5X and indels were excluded. This resulted in a high-quality dataset containing 230,597 SNPs.

The Colonial strains from Mexico, Tepos-20 and Tepos-37 were not considered due to their low coverage; and Tepos-10 was also discarded due to its high heterozygosity levels. Although it did not reach the coverage thresholds, the la Sagrera genome was used in the subsequent analysis after undergoing strict filtering. We compared different filtering parameters to find a balance between covered positions and possible erroneous calling (Figure S8). Accordingly, we decided to trim 10 bases from the ends of each of the DNA reads using *TrimBam* (Jun et al., 2015). Following this step all positions in the la Sagrera sample were called with the reference allele considered as valid independent of their coverage. For variant positions, only SNPs found in the high-quality dataset were considered independently again of their coverage; all other positions were discarded.

#### Maximum likelihood tree

All positions satisfying the filtering criteria were used to create a consensus genome *fasta* using the reference genome as template. Those positions that were filtered out in the previous steps were masked in the resultant fasta file. This resulted in a phylogenetic dataset comprising 414 *Salmonella enterica* AESB and an outgroup (ERS217420).

An initial Maximum Likelihood (ML) tree was built using *RAxML* with the nucleotide substitution model GTRCAT and a strain of *Salmonella enterica* ser. Enteriditis as an outgroup. The resultant tree was visualized using the R package *ggtree* and *iTOL* (Stamatakis, 2014; Yu et al., 2017; Letunic and Bork, 2019). Following this, we applied *ClonalFrameML*, with default parameters, to correct the subsequent tree for the presence of homologous recombination events (Didelot et al., 2015). *ClonalFrameML* can infer the location of a recombination event in each branch of a ML tree, which can impact the resultant terminal branch lengths. Additionally, *ClonalFrameML* imputes missing data from the input sequences. We particularly analyzed the presence of recombination events in the Paratyphi C subtree. The transition / transversion ratio used (κ) was 2.097.

After removing the recombination events present in the Paratyphi C lineage, we proceeded to remove all remaining homoplasies from the dataset using *HomoplasyFinder* (Crispell et al., 2019). A homoplasy is defined as a substitution event which has arisen independently in different separate lineages, and which can alter the tree topology, hence these may arise from undetected recombination or spurious variant calls which may appear in low coverage samples. Homoplasies are determined by their consistency index value which ranges between 0 and 1, with values closer to zero denoting more homoplasic variants (Fitch, 1971). A final ML tree, based on the filtered alignment of 3680 variant sites, was generated using *RAxML* with the parameters described above. As before, the Paratyphi C phylogeny was visualized using *ggtree* (Yu et al., 2017).

#### Temporal signal exploration

In order to perform phylogenetic tip-calibration it is necessary to first confirm the presence of significant temporal signal in the alignment. Specifically, we tested the Paratyphi C branch of the phylogeny. A temporal regression of sampling date (years) against root-to-tip distance was performed using the R package *BactDating*, which additionally conducts a date randomization test of significance (Didelot et al., 2018). Samples without an assigned collection date were treated as missing. We established the most suitable model by running all models and comparing their *DIC* and *ESS* value (Plummer et al., 2006; Didelot et al., 2018) (Table S8). We performed the analysis using the model *arc* inference model and 1·10^7^ iterations. The resulting time calibrated phylogeny was visualized using *ggtree* (Table S9).

### Quantification and statistical analysis

Quantification of model statistics were performed in R v4.1 using the packages CODA and BactDating. Lowest DIC values and highest ESS values were prioritized. A *p-value* of ≤ 0.05 was considered statistically significant.

## Data Availability

• All bacterial and human shotgun sequencing data have been deposited at ENA (project PRJEB43276) and are publicly available as of the date of publication. Accession numbers are listed in the key resources table. • This paper does not report original code. • Any additional information required to reanalyze the data reported in this paper is available from the lead contact upon request.

## Supplemental information

Document S1. Figures S1–S8 and Tables S1, S2, S3, and S5

Table S4. Reference mapping statistics for a diverse set of 839 *Salmonella enterica* assemblies used to identify the closest serovar to F1691-1810, Related to Figure 2 and Table S3

Table S6. Coverage over virulence genes identified in Paratyphi C, Related to Figure 2 and Figure S2

Table S7. Recombination events detected in the Paratyphi C alignment by ClonalFrameML, Related to Figure 3

Table S8. Comparison of the temporal estimates obtained when applying different phylogenetic tip-dating models implemented in BactDating, Related to Figure 4

Table S9. Estimated divergence dates in the Paratyphi C phylogeny, Related to Figure 4
