# Supplementary material for: Salmonella enterica from a soldier from the 1652 siege of Barcelona (Spain) supports historical transatlantic epidemic contacts
Source: iScience. 2021 Aug 24;24(9):103021. doi: 10.1016/j.isci.2021.103021 (PMC8430385; doi:10.1016/j.isci.2021.103021)
Supplement: Document S1. Figures S1–S8 and Tables S1, S2, S3, and S5 [file mmc1.pdf]

iScience, Volume *24*

Supplemental information

Salmonella enterica from a soldier

from the 1652 siege of Barcelona (Spain) supports

historical transatlantic epidemic contacts

Toni de-Dios, Pablo Carrión, Iñigo Olalde, Laia Llovera Nadal, Esther Lizano, Dídac Pàmies, Tomas Marques-Bonet, François Balloux, Lucy van Dorp, and Carles Lalueza-Fox

Supplementary Materials

***Salmonella enterica* from a soldier from the 1652-siege of Barcelona (Spain) supports historical transatlantic epidemic contacts**

Toni de-Dios, Pablo Carrión, Iñigo Olalde, Laia Llovera Nadal, Esther Lizano, Dídac Pàmies, Tomas Marques-Bonet, François Balloux, Lucy van Dorp, Carles Lalueza-Fox

Supplementary Tables

Sample Name	nºSequenced PairedReads	Unique Reads	Q30 Reads	Average Coverage	Genetic Sex	X Chr Contaminatio n	SE
**F1364_1436**	99,018,404	3,014,847	2,906,004	0.0997X	XY	3.55%	4,49%
**F1691_1810 March**	97,403,676	2,138,596	2,054,187	0.0688X	XY	9.30%	9,34E-02
**F1691_1810 October**	149,069,621	1,280,032	1,196,432	0.039X	XY	Not detectable	-

Table S1. **Human mapping statistics, related to Figure 1.** Mapping statistics of the analysed samples against the human reference genome hg19.

Sample_Name	Q30 Reads	Avr.Depth	Majoritary Haplogroup	Minoritary Haplogroup	Contamination Es.Schmutzi
**F1364_1436**	3,768	22.69	H2a5a	-	Mean:0;Lower:0;Upper:6%
**F1691_1810 March**	5,178	32.86	T2b	U5b1f1a	Mean:0;Lower:0;Upper:95%
**F1691_1810 October**	15,275	129.77	U5b1f1a	-	Mean:0;Lower:0;Upper:0.5%

Table S2. **Mitochondrial mapping statistics, related to Figure 1.** Mitochondrial mapping statistics, haplogroup determination and contamination estimates.

KRAKEN2				
**Sample**	**Species**	**Summative Taxon Reads**	**Exact Taxon Reads**	**% of all Reads in sample**
**F1364-1436**	*Yersinia* (genera)	3,343	363	0.0034%
	Y. enterocolitica	244	175	0.000246419
	Y. pestis	3	2	3.03E-06
	Y. similis	34	34	3.43E-05
	Y. pseudotuberculosis	1,827	169	0.001845112
	S. enterica	1,398	681	1.51E-02
**F1691-1810**	*Yersinia* (genera)	6,127	1,401	0.0025%
	Y. enterocolitica	447	361	0.000181358
	Y. pestis	75	63	3.04E-05
	Y. similis	138	138	5.60E-05
	Y. pseudotuberculosis	945	177	0.000383409
	S. enterica	14,326	9,006	0.01%
**BLAST**				
**Individual**	**Specie**	**Mapped**	**Unique**	**Blast hit**
**F1364-1436**	*Enterocolitica*	286	269	5
	Pestis	282	268	4
	Pseudotuberculosis	281	268	4
	Similis	281	264	4
**F1691-1810**	*Enterocolitica*	532	501	9
	Pestis	505	462	8
	Pseudotuberculosis	517	474	8
	Similis	551	503	13

Table S3. **Metagenomic assignation summary, related to Figure 2**. Metagenomic read assignation to *Yersinia* species and *Salmonella enterica* using kraken2 and BLAST.

Sample Name	Sequenced Paired Reads	Mapped reads	Unique Reads	Quality 25 Reads	Average Depth of Coverage	% of Covered Positions	%duplication mapped reads
**F1364-1436**	99,018,404	1,650	1,572	1,452	0.003X	0.006%	4,72%
**F1691-1810**	246,473,297	36,051	24,327	24,225	0.3852X	30.78%	32,52%

Table S5. **Salmonella mapping statistics, related to Figure 2.** Mapping statistics of the analysed samples against the *Salmonella* Paratyphi C reference genome.

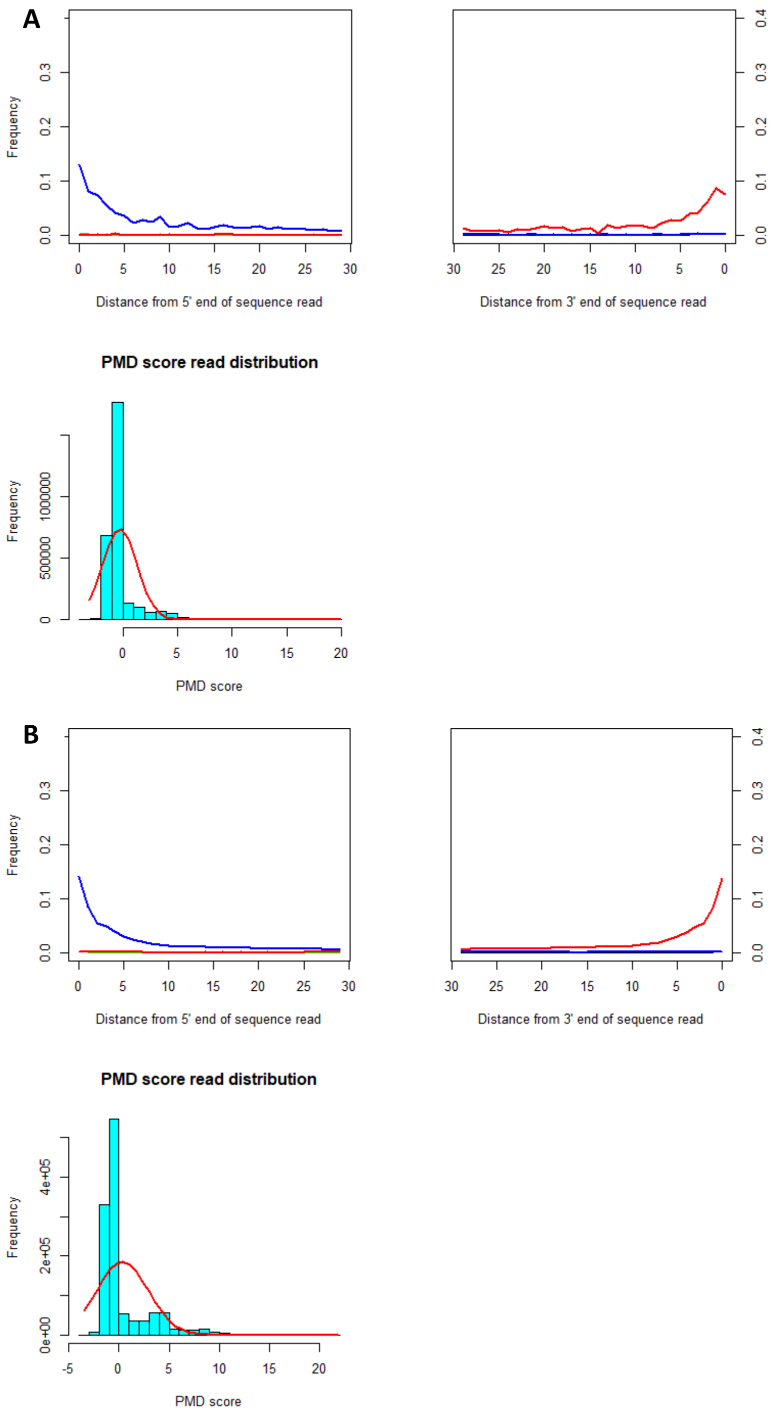

Figure S1. **Human aDNA damage patterns, related to Figure 1**. Ancient DNA authenticity damage patterns observed in human reads from F1691-1810 (A - top) and F1364-1436 (B - bottom).

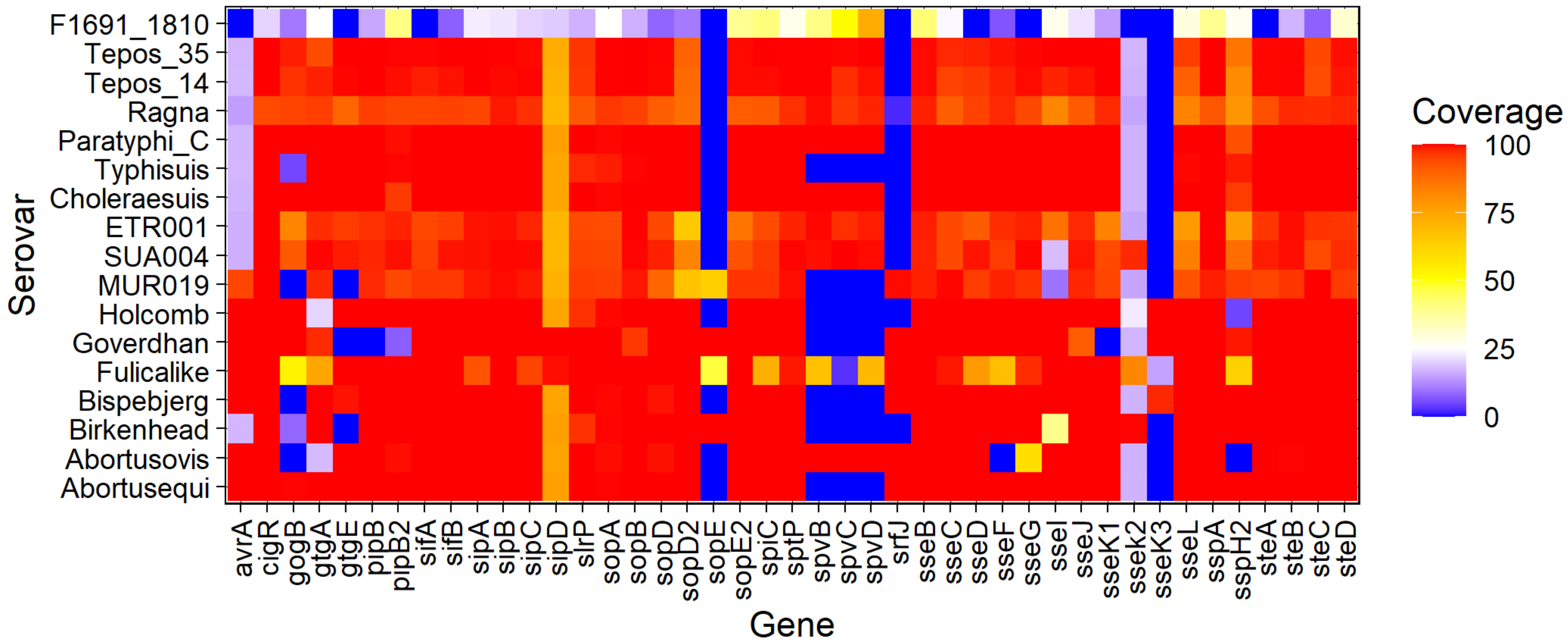

Figure S2. **Salmonella virulence gene presence, related to Figure 2.** Heatmap displaying the % of positions covered by at least one depth of coverage in a set virulence associated gene. Due to the low coverage of the analysed Sagrera (F1691-1810) strain, the absence of genes cannot be properly demonstrated.

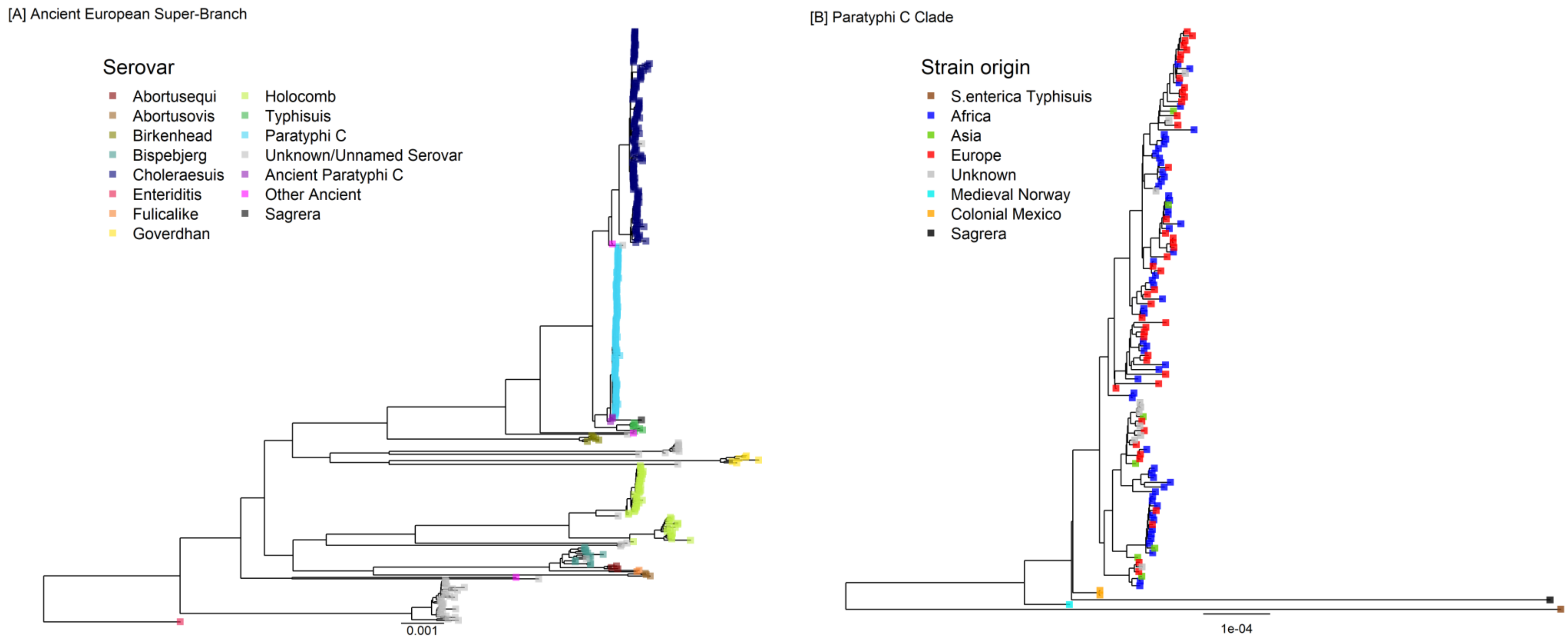

Figure S3. **Exploratory phylogeny of La Sagrera, related to Figure 3.** Maximum likelihood phylogeny of *S. enterica* falling within the Ancient Eurasian Super Branch (AESB). (A) ML tree of La Sagrera and 413 S. enterica strains representing the ancient and modern diversity of the AESB from previous studies (Vågene et al., 2018; Zhou et al., 2018; Key et al., 2020). The tree is rooted using S. enterica ser. Enteriditis from a previous study (Alikhan et al., 2018). Serovars are coloured at the branch tip. (B) Maximum likelihood phylogeny of the Paratyphi C clade including 124 Paratyphi C modern and ancient strains. The tree includes La Sagrera strain, 119 Modern Paratyphi C strains (Key et al., 2020), 2 strains from Colonial Mexico (Vågene et al., 2018), a strain from Medieval Norway (Zhou et al., 2018) and a S. enterica ser. Typhisuis as an outgroup (Key et al., 2020).

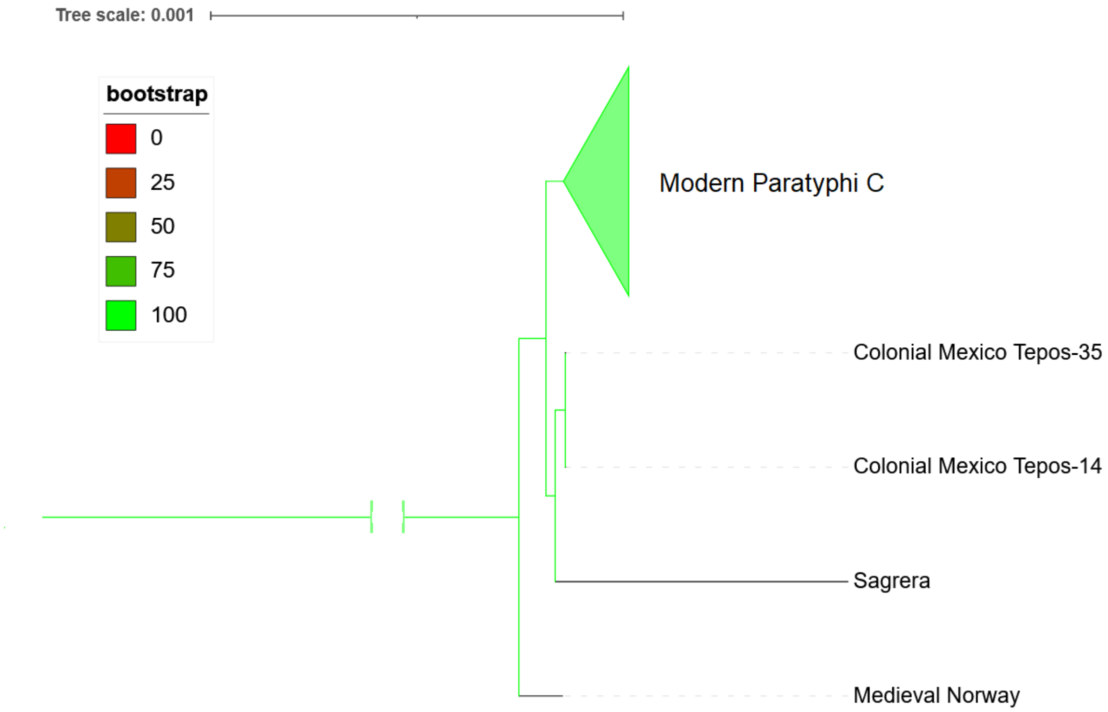

Figure S4. **Paratyphi C clade bootstrap support, related to Figure 3.** Zoom-in of the Paratyphi C sub-branch. Bootstrap values are displayed using a colour key. Modern strains are collapsed for easier visualisation. Ancient strains, including la Sagrera, have a high node support (100%) in the ML phylogenetic tree.

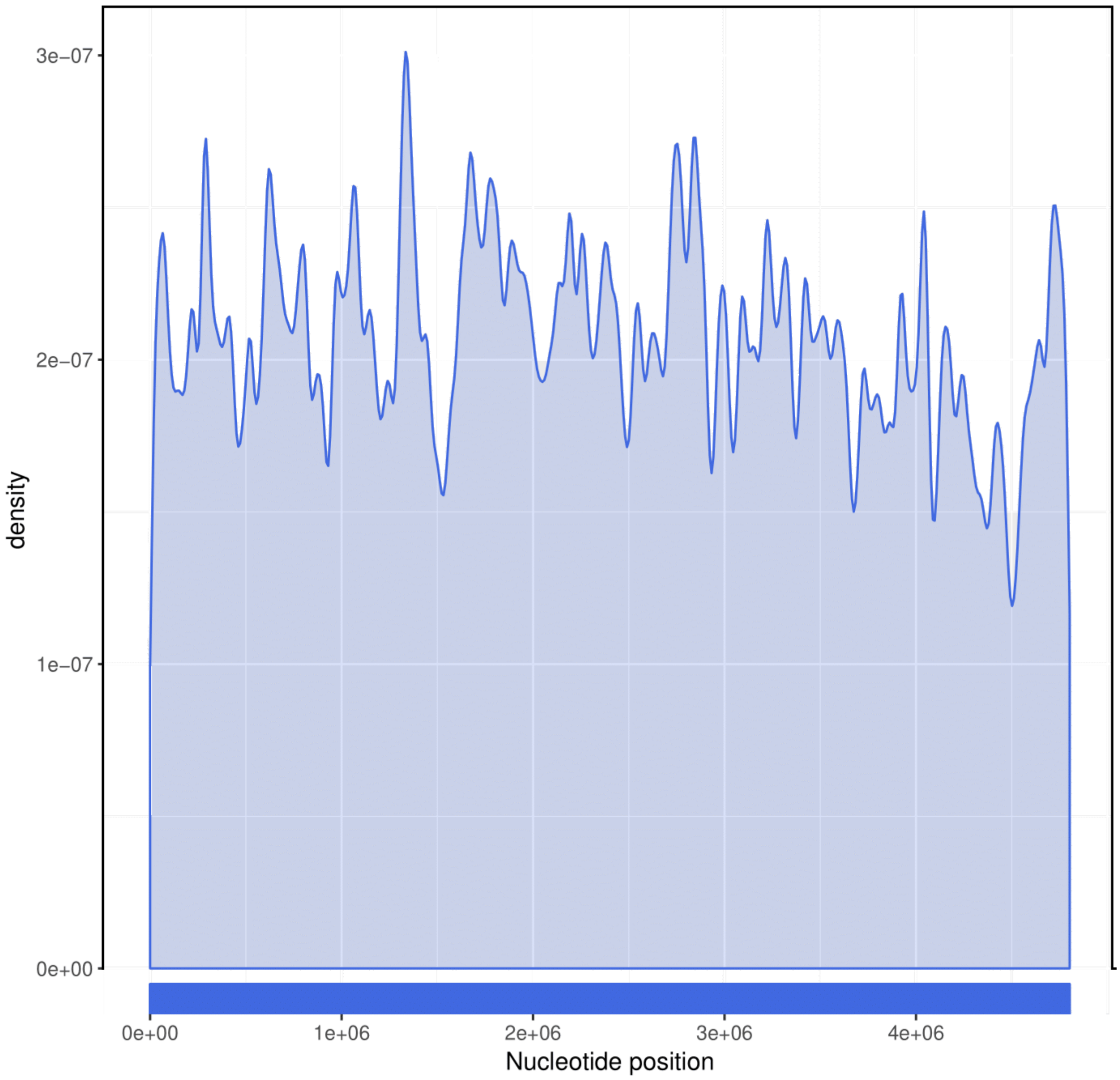

Figure S5. **Paratyphi C SNP distribution, related to Figure 3**. Distribution of the 4,098 SNPs remaining along the *Paratyphi C* genome after the removal of putative recombination events inferred by ClonalFrameML. This dataset represents roughly 30% of the original alignment.

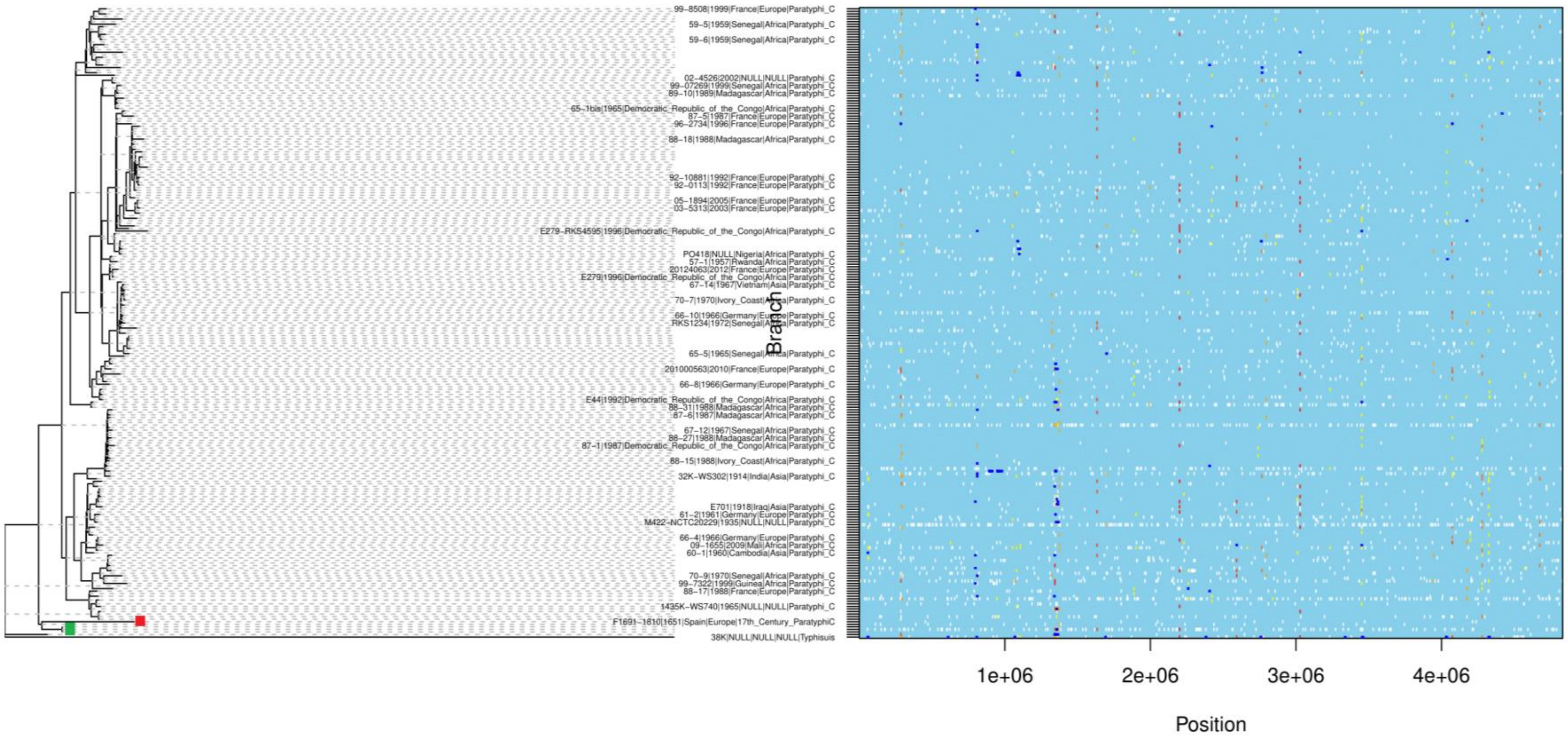

Figure S6. **Paratyphi C recombination event distribution, related to Figure 3**. ClonalFrameML inferred recombinant tracts with recombination corrected ML tree of *S. enterica* AESB. La Sagrera and the Colonial Mexico tips are highlighted in red and green respectively. The heatmap at right provides a representation of genomic events along the Paratyphi C chromosome. Recombination tracts are marked as dark blue bars. White bars give non-homoplasic substitutions, while the bars ranging from yellow to red represent homoplasic sites (with a red value denoting a more homoplasic position). The tree includes La Sagrera *Salmonella* Paratyphi C, 2 Colonial Mexico strains from Vågene et al. (2018); a Medieval strain from Norway from Zhou et al. (2018); 119 Modern Paratyphi C strains and a modern Typhisuis strain from Key et al. (2020) as an outgroup.

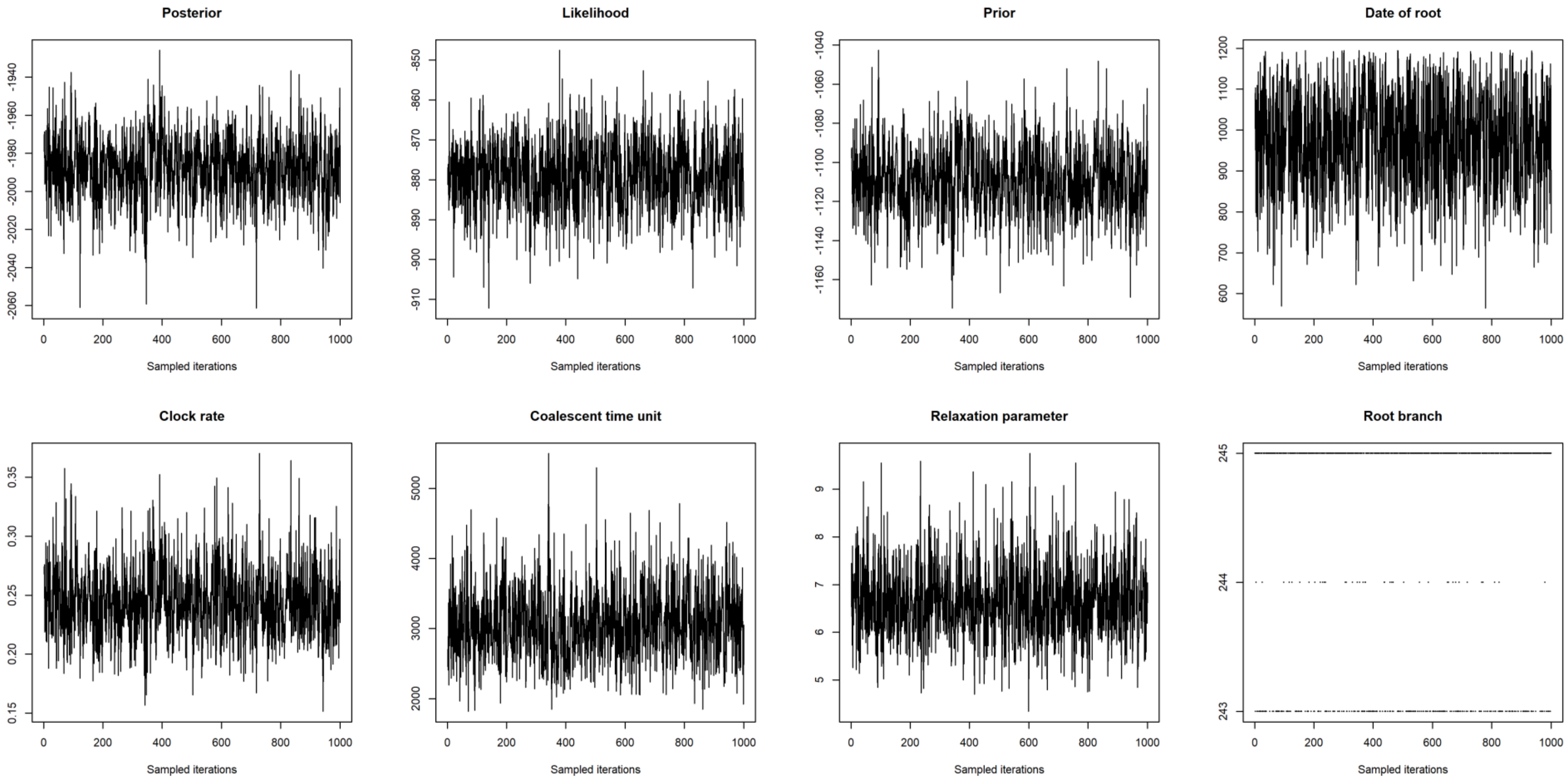

Figure S7: **BactDating convergence traces, related to Figure 4.** MCMC convergence traces for key phylogenetic parameters following $1 \cdot 10^7$ iterations of BactDating.

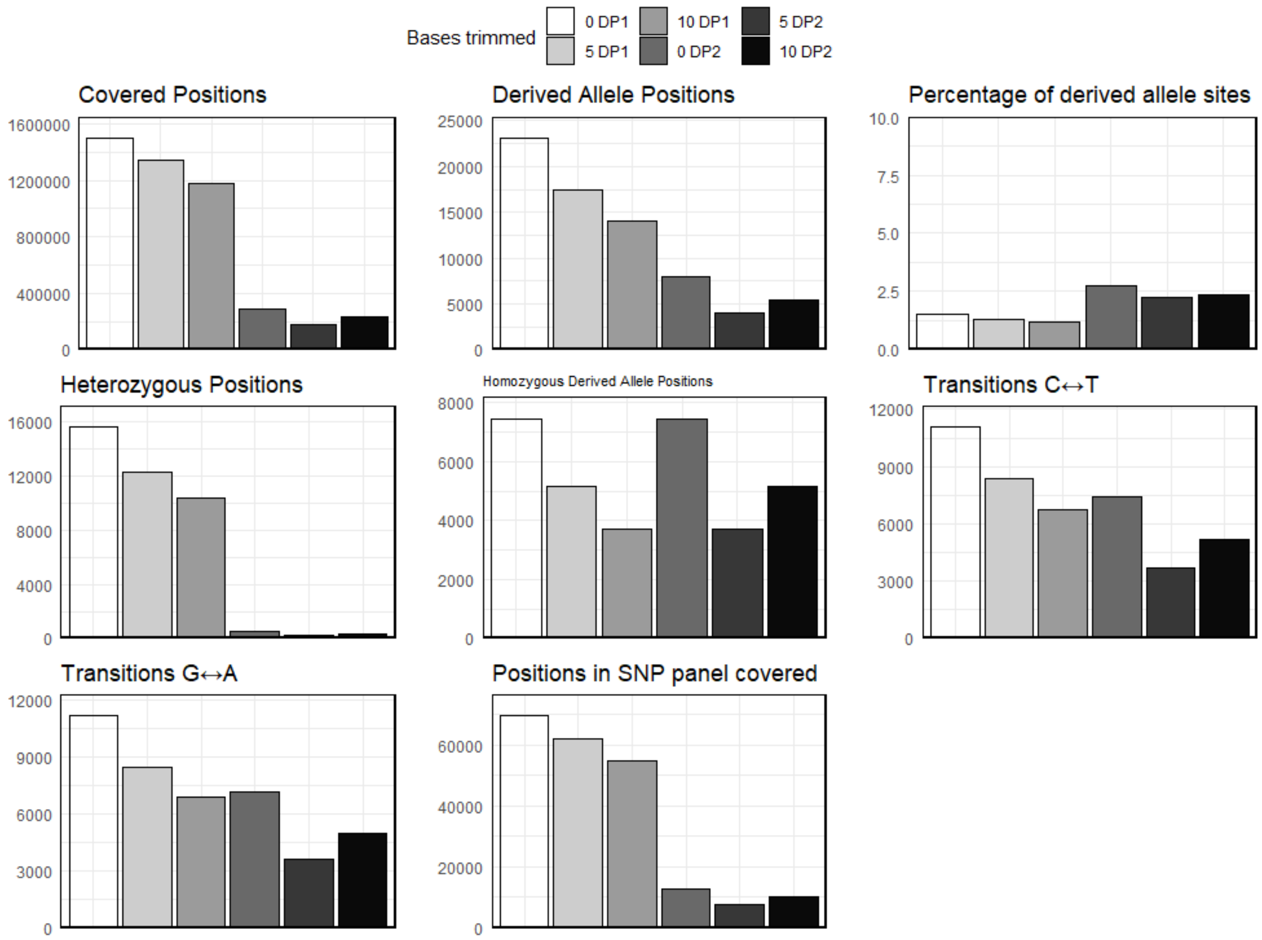

Figure S8: **Filtering parameters comparison, related to Figure 3**. Effects of different filtering parameters on variant calling statistics in our Paratyphi C sample. Using a minimum allele depth of 2 removed most of heterozygous positions, which were already excluded by choices within our selected pipeline. Filtering for depth drastically impacts the coverage of the SNP panel. We note that trimming effectively reduce the number of transversions and positions with a derived allele call while maintaining a more or less stable number of covered positions in the high-quality SNP panel.
